# Influence of 2 Digital Exercise Modules of a Multimodular System on Balance and Leg Strength Under Consideration of Use Adherence: Prospective Cohort Study

**DOI:** 10.2196/36805

**Published:** 2022-09-19

**Authors:** Verena Venek, Christina Kranzinger, Sonja Jungreitmayr, Susanne Ring-Dimitriou, Hermann Schwameder, Thomas Stöggl

**Affiliations:** 1 Salzburg Research Forschungsgesellschaft mbH Salzburg Austria; 2 Department of Sport and Exercise Science Paris Lodron University Salzburg Salzburg Austria; 3 Red Bull Athletes Performance Center Salzburg Austria

**Keywords:** active and assisted living, functional fitness training, information and communication technology, use adherence

## Abstract

**Background:**

To empower healthy aging, digital solutions embed multiple modules for physical activity, cognitive health promotion, and social engagement. Integrating new empowering technologies such as digital exercise monitoring requires assessment measures and analysis procedures, considering variable compliance of users with different modules.

**Objective:**

This study aims to assess the influence of a tablet-based and a feedback system–based exercise module on balance and leg strength by considering use adherence instead of the use of the entire multimodular system.

**Methods:**

In the prospective cohort study within the fit4AAL project, 83 users (n=67, 81% women; n=16, 19% men; mean age 66.2, SD 2.3 years) used the 2 digital exercise modules of a multimodular physical activity promotion system for >18 weeks. A data-driven clustering method based on the average use frequency of the exercise modules determined the number of user types that met the World Health Organization–recommended training frequency of at least twice per week. On the basis of this use adherence, statistical analysis was performed with features of functional performance tests (unipedal stance, 30-second chair rise, Y-balance, and hurdle step tests). The tests were conducted 6 months before the intervention, immediately before the intervention, and after the intervention, comparing the baseline phase with the 3 feedback use groups of the study (using only the tablet, the tablet and the feedback system, or only the feedback system).

**Results:**

Of the 83 users, 43 (52%) met the World Health Organization–recommended frequency of muscle-strengthening activities. Overall, the feedback use groups achieved, on average, more chair rises in 30 seconds than the baseline group (*P*=.01; moderate effect size of 0.07). Of the 43 users, 26 (60%) additionally used the feedback system–based exercise module. They improved in balance compared with the users using either the tablet or the feedback system (*P*=.02). In addition, they improved their leg strength within the group (*P*=.04) and compared with the baseline (*P*=.01).

**Conclusions:**

The additional use of a feedback system showed a tendency to positively maintain and influence the already exceptionally high functional performance of older adults. Considering use adherence in future multimodular system studies is crucial to assess the influence of single and combined use of exercise modules on functional performance.

## Introduction

The things we do and value in life affect how we age. The ultimate goal is to age healthily and, therefore, increase the number of healthy years of life. The World Health Organization (WHO) describes healthy aging as “the process of developing and maintaining the functional ability that enables wellbeing in older age” [1]. Functional ability encompasses the intrinsic capacity of people’s mental and physical abilities to cope with daily life. Physical abilities affect mental abilities and vice versa [2]. In the field of health promotion, physical activity has been prescribed as a preventive measure for reducing risks of functional ability and noncommunicable diseases [3].

To engage older people and promote physical activity, new empowering technologies have been considered to support compliance with training at home. In comparison with assistive technologies, which aim to alleviate the effects of disabilities such as immobility, empowering technologies aim to help people prevent functional disability [4]. For the users, it has to be an experience to maintain or even increase compliance with exercise and make the long-term effects tangible. Therefore, digital exercise modules have included different technologies and devices, such as fitness trackers and camera systems, to provide feedback on exercise performance and movement quality during training sessions.

Although interventions with exercise feedback and monitoring have been used with positive results on functional performance, procedures to thoroughly report and test the digital interventions and their adherence for older adults are lacking [5]. This assessment is even more challenging when, in particular, older participants train unsupervised [6]. Nevertheless, this setting is common in the field of Active and Assisted Living (AAL). For example, AAL projects funded by the AAL Joint Programme of the European Union should integrate a proof of concept, market potential, and strategies of the solutions, as well as impact evaluations, into 3- to 4-year AAL projects [7]. Hence, studies evaluating the effects of technology-assisted physical activity interventions in real-life settings are required [8,9].

The goal of AAL research is to promote active and healthy aging by developing and investigating age-appropriate services and solutions based on Information and Communication Technology (ICT). The developed solutions are often ICT–based multimodular systems that address ≥1 component of successful aging; that is, chronic disease management, maintenance of physical and cognitive health, and active social engagement [10]. For physical health, the WHO recommends regular muscle-strengthening training involving major muscle groups for the age group of 18 to 64 years on ≥2 days a week [11]. To achieve the recommended training frequency, digital exercise modules integrated into multimodular solutions have been considered to augment usual coaching, reduce costs, and increase training accessibility to support people in establishing training routines [12-14].

Despite proven long-term effects of regular exercise on functional ability, the impact measures and analysis procedures for evidence of the usefulness of digital exercise modules vary. Although the use adherence in the ZentrAAL project had been analyzed independently from functional performance [15], CareInMovement introduced an expert-based clustering of user types to identify use-related differences [16]. However, the workouts provided in both projects were accessible via a tablet app, and the effects on participants’ functional performance related to the fitness program were investigated rather than the impact of the technological devices themselves on physical capabilities. Dasgupta et al [10] surveyed tablet-based intervention studies, which were characterized by limited sample sizes and durations, and revealed significant improvements in functional performance in terms of gait velocity, repeated chair rises, and static balance. However, the mentioned apps mainly focused on providing training content without feedback or monitoring exercise execution.

A study protocol given by Belleville et al [17] described the intended outcome analysis for the tablet-based multimodular solution *StayFitLonger* related to physical and cognitive health training at home. Performance on the “Timed-Up & Go” test was selected as the primary outcome measure of physical health, which is assessed before and after the 26-week intervention. Use adherence was mentioned to be considered in future evaluations to determine whether the recommended dose, volume, and frequency of the physical activity program could be maintained over time and to evaluate the efficacy.

A similar multimodular AAL solution for physical activity promotion was developed in the Austrian AAL project, fit4AAL. The purpose of the modules was not only to support daily physical activity and regular exercising but also to promote knowledge about an active and assisted lifestyle. The fit4AAL field trial included 2 phases using a randomized controlled trial design with a waitlist baseline group [18]. In the first trial phase with a randomized-assigned intervention group and a baseline group, the impact of the entire solution was investigated. For example, the exercise modules improved muscular strength and flexibility in older women in the first phase [19]. In the second trial phase, the same but matured digital intervention was applied to the baseline group. In both phases, the AAL solution integrated the 2 digital exercise modules. A tablet-based version and a feedback system–based version of a personalized training program focused on improving functional abilities and establishing a training routine.

To evaluate the influence of the digital exercise modules of the multimodular solution on balance and leg strength, including the movement quality of older adults, we hypothesized that the additional use of the feedback system–based exercise module would improve the functional performance of the participants when they met the WHO-recommended training frequency. As the feedback system–based exercise module was more mature in the second phase of the field trial, this prospective cohort study investigated the data from the participants of the waitlist baseline group of the fit4AAL field trial. Moreover, one of the questions was how many participants met the WHO-recommended weekly training frequency during the intervention phase. Therefore, use adherence was used to investigate the compliance with the 2 exercise modules.

## Methods

### Digital Exercise Modules and Training Program

The multimodular AAL solution comprised 4 modules: an e-learning module, an activity-tracking module, and 2 exercise modules forming the digital home training. One of the two personalized digital exercise modules was accessible via a tablet-based Android app version, including the other modules as well. The other exercise module provided live feedback for older adults during training via the feedback system–based Android app version. This feedback system monitored exercise through skeleton tracking of the 3D camera system Orbbec Persee (Orbbec) connected to the users’ television monitors. This version, as well as the tablet-based exercise module, offers training sessions tailored to users’ fitness levels, with 365 functional exercises [20].

The used multidimensional training programs focused on varied but structured movements to maintain or even increase functional fitness [21]. All training sessions comprised a warm-up, main, and cool-down phase. Exercises to mobilize and invigorate the cardiovascular system, such as shoulder circles and marching in place, started the warm-up. The main part included strengthening exercises such as squats and table push-ups. Stretching exercises for the lower and upper body concluded each training session. The users could select among 10-minute, 20-minute, or 30-minute training sessions, and the suggested workouts changed daily. At the start of each exercise, a thorough description of the exercise in written, video-based, and spoken form was available.

The modules differed after the exercise description. In the exercise module on the tablet, the exercise description video and a countdown were displayed while the users were performing the exercise for the prescribed amount of time. For each exercise, users had the possibility to either skip the exercise or confirm it by clicking on the corresponding buttons in the app. In both cases, the next exercise was described. After the last exercise, an overview of the workout, including the actual workout duration and number of performed exercises, was displayed.

**Figure 1:**
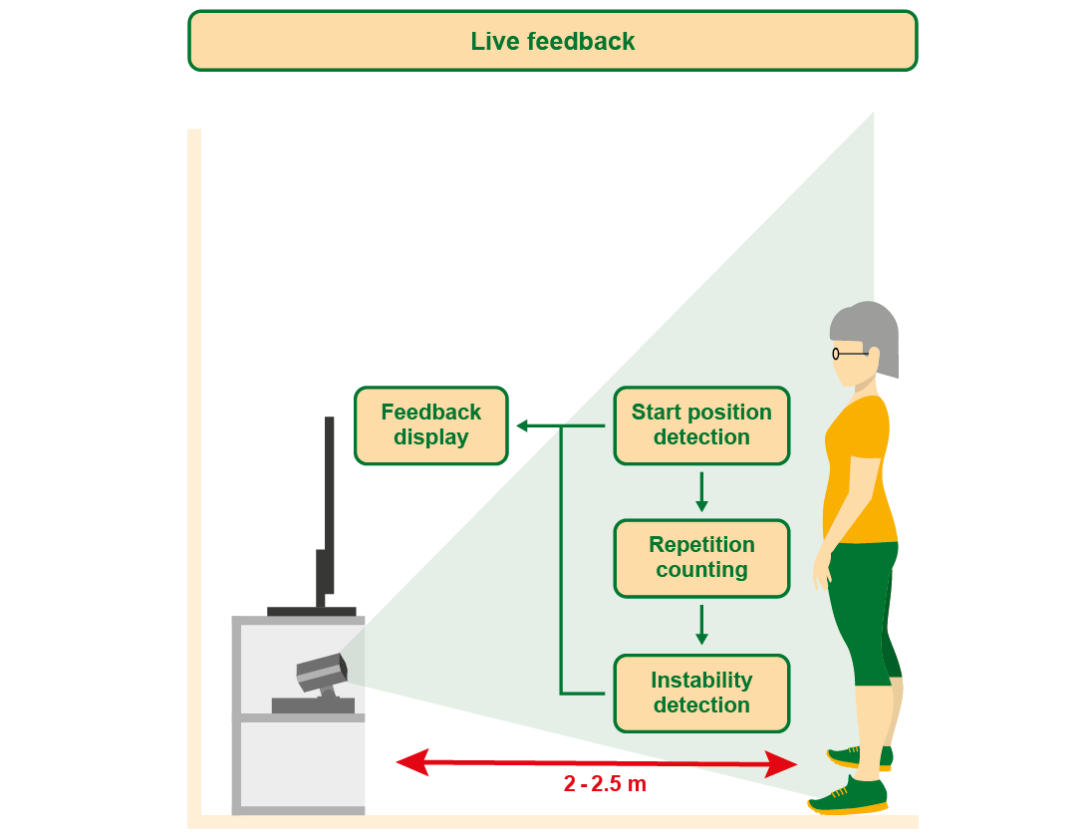
Live feedback- and exercise-monitoring functionalities of the feedback system–based exercise module and recommended distance from the 3D camera system of 2 to 2.5 meters.

Additional live feedback during exercise execution was available in the feedback system–based exercise module. To use skeleton tracking (Nuitrack SDK, version 1.3.1; 3DiVi Inc), the users were advised to stand 2 to 2.5 meters away and in front of the Persee (Figure 1). In addition to the exercise description video, a camera live stream mirrored users so that they could visually adjust and correct their posture. Furthermore, several exercises were tagged with feedback-providing algorithms: from the 365 exercises, 154 were tagged with one of the start position detection algorithms, 122 were tagged with one of the repetition counting algorithms, and 100 were tagged with the instability detection algorithms.

Repetition counting and instability detection required start position detection. Start position detection algorithms supported users in taking the required pose for an exercise. For example, squats were counted only when users took the required starting position of hip-wide standing. Repetition counting algorithms enabled the users to follow the number of repetitions they achieved, such as the number of squats they performed. Additional instability detection algorithms monitored the correct postures during exercise execution. For example, during the one-leg stand exercises, the users were visually notified when their sway to either side left a balanced stance. As the skeleton tracking of the 3D camera system was used, the focus was on exercises starting from the standing or sitting position [22]. This excluded exercises in prone or supine positions performed on the floor or leaning against a wall, as well as exercises where users faced the camera laterally. The camera live stream in the app was available for all exercises.

### Participants

The thorough recruitment process of fit4AAL participants in Austria was described by Trukeschitz et al [18]. It was separated into three stages: potential participants (1) received letters of invitation via mail, magazines, newspapers, websites, and digital newsletters; (2) had to confirm their interest following a developed questionnaire on the web or by telephone; and (3) were selected by the project team based on the questionnaire answers. The questionnaire was used to identify individuals who had been retired for 2.5 to 6 years and were willing to participate in a scientific study of physical activity promotion, were not dependent on mobility aids, had no chronic diseases or physical limitations, did not have a personal trainer at the time of recruitment, or exercised >4 times per week. Furthermore, participants who were technology savvy were selected based on possession of an email address, a monitor, and free space (approximately 2-2.5 meters) in front of the monitor to exercise.

**Figure 2:**
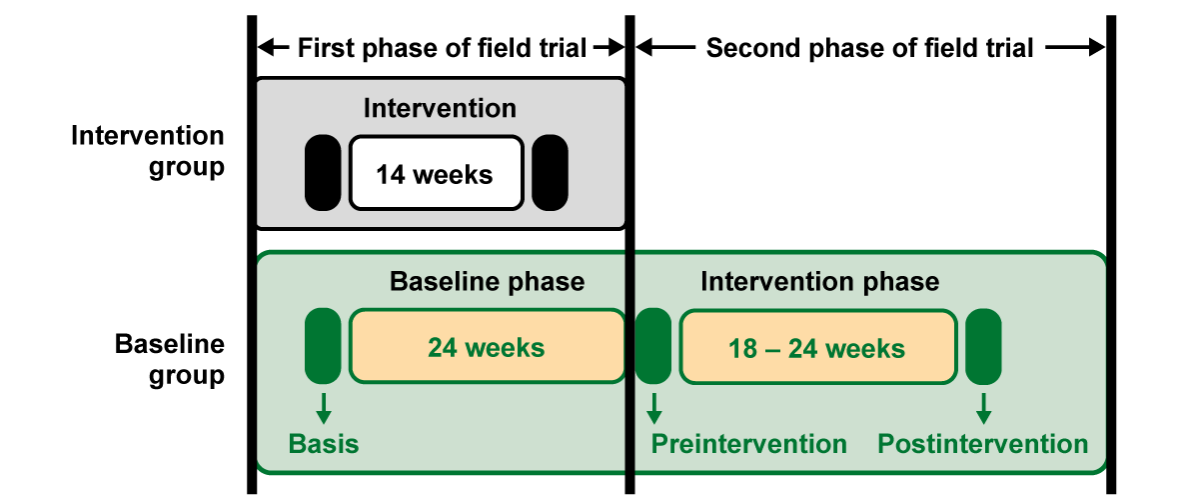
Field trial phases with a randomly assigned intervention group and waitlist baseline group. The trial phases of the baseline group were separated into baseline and intervention phases, including 3 functional performance assessments (basis, preintervention, and postintervention).

The selected study participants were randomly assigned to the intervention and baseline groups of the first fit4AAL trial phase. The baseline group of the first trial phase became the intervention group of the second trial phase because of the randomized controlled trial with a waitlist baseline group design [15]. This means that the multimodular AAL solution was first applied to the intervention group and then to the baseline group. For the prospective cohort study of the baseline group, the baseline and intervention phases of the baseline group were investigated (Figure 2).

Excluding dropouts and nonusers from the 109 participating adults, 91 (83%) used at least one of the four modules of the multimodular AAL solution during the intervention phase [23]. Of the 91 users, 86 (94%) used either 1 of the 2 or both digital exercise modules. This resulted in an adherence rate of 94.5% to the exercise modules of the AAL solution. From these 86 users, 3 (3%) were removed as they did not show up for the tests or they had not finished any workout. The average age of the 83 users (n=67, 81% women and n=16, 19% men) was 66.2 (SD 2.3) years.

### Ethics Approval

All study participants were informed about their rights, data use, and exit strategies by signing an informed consent form before the study. The study design was positively evaluated by the ethics committee of the University of Salzburg (EK-GZ: 09/2018). The videos of the feedback system–based module were neither saved nor sent to any servers to ensure the participants’ privacy. The skeleton tracking was performed on the depth images exclusively on the system and did not require any video data.

### Study Design

A prospective cohort study was conducted within a year. Participants performed functional performance tests supervised by sports scientists for basis and pre- and postintervention measurements. The functional performance tests included the unipedal stance (UPS) test, the 30-second chair rise test (CRT), the Y-balance test (YBT), and the hurdle step (HS) test.

Furthermore, 6 months before the intervention, the basis measurements were collected within 4 to 8 weeks because of staged appointment coordination. The data from the basis and preintervention assessment points defined the baseline phase. The preintervention functional tests assessed the fitness level of the participants for the configuration of the training program. For the following 18 to 24 weeks, the participants received the multimodular AAL solution with the digital exercise modules for their homes. After the intervention, the participants returned to the sports scientists for the postintervention measurements. Figure 2 shows the timeline of the study.

During the intervention phase, the workout information and app use were monitored. The workout data comprised the workout duration in hours, the workout duration performed with the feedback system, and the number of completed workouts. Furthermore, the workout data included the number of times the 10-minute, 20-minute, or 30-minute workouts were selected.

App use data were recorded by digitally logging the visits of the modules of the AAL solution via the open-source web analytics tool (Matomo, InnoCraft). A visit was defined as continuous viewing of either one of the exercise modules without breaks for >30 minutes. Aggregated use data included information on visits per day and whether users viewed either the tablet-based or the feedback system–based exercise module.

### Descriptive Analysis of Use Adherence and Feedback Use Groups

Voluntary use at home was expected to influence the compliance with the modules, as well as with the exercise modules. Thus, use adherence was defined in visits to either one of the two exercise modules, determining how often the participants used the exercise modules on average during the intervention. To determine the user types, the Jenks natural breaks cluster algorithm was applied based on the average frequency of visits to the exercise modules to define the interval limits, indicating the use adherence of different user types to the exercise modules in visits per day. The limits multiplied by 7 days estimated the number of visits per week.

The statistical analysis required the distribution of users to the different versions of the exercise modules, resulting in 3 feedback use groups: they described the number of users who trained only with the tablet-based exercise module, those who trained additionally with the feedback system–based exercise module, and those who trained only with the feedback system. For each of the 3 feedback use groups, descriptive statistics, including age, sex, average workout data, and baseline test results, were determined. The reported sex categories were male and female. In addition, the user types with upper interval limits smaller than 2 visits per week were rejected as they did not meet the WHO-recommended amount of muscle-strengthening activities of at least twice per week. Thus, descriptive statistics and statistical analysis were repeated for the adapted feedback use groups, considering use adherence.

### Outcome Measures

#### Balance

The UPS test represents and assesses static balance by recording the time in seconds the participant is able to stand on one leg without relying on the standing leg [24,25]. The maximal duration of the UPS was set to 60 seconds and the times were noted in seconds to the nearest tenth. Of the 3 attempts for each leg, the maximal durations for both legs (UPS maximum) and each leg were determined (UPS left leg maximum and UPS right leg maximum).

#### Leg Strength

The CRT represents and assesses the lower body strength by counting the number of chair rises that can be completed within 30 seconds [26]. A correct chair rise starts with the participant sitting on the chair with arms crossed at chest height, shoulder wide stance, and feet in full contact with the ground, and it ends in a standing position with the hips and knees fully extended.

#### Balance and Leg Strength

The YBT assesses the stability, range of motion, strength coordination, and mobility of the upper and lower body for both the left and right sides [27]. It represents dynamic balance and leg strength. The composite reach distances or composite scores for the left leg (YBT left leg) and right leg (YBT right leg) are the sums of the 3 reach directions (anterior, posteromedial, and posterolateral) divided by 3 times the limb length. Participants had to maintain balance on their dominant leg while they were encouraged to reach indicators in the 3 directions as far as possible. Furthermore, a leg symmetry index (YBT leg symmetry) was determined by calculating the difference between the composite reach distance of the left and right leg.

#### Movement Quality

The HS is 1 of the 7 fundamental movement patterns of the Functional Movement Screen tests and assesses the functional symmetry by scoring the performed HS representing a measure of human movement quality related to balance and leg strength [28]. The categorical scores were 1, 2, or 3, with a score of 3 expressing the best performance and a score of 1 expressing the worst. The HS test was attempted 3 times, with the highest scores used for the analysis for each leg (HS left leg and HS right leg).

### Statistical Analysis

Statistical analyses were performed using the statistical software R (version 4.0.3; R Foundation for Statistical Computing) [29] and the *rstatix* (version 0.6.0) package [30]. The significance level was set to α<.05. To determine significance within the groups, a paired Wilcoxon signed-rank sum test was used on the pre- and postintervention data of the feedback use groups and on the basis and preintervention data from the baseline group.

To assess the influence of the training with the digital exercise modules on balance and leg strength, we used the difference between pre- and postintervention measurements from the feedback use groups and the difference between basis and preintervention measurements as control. The Shapiro-Wilk test was used to screen for normality. As the data set was not normally distributed, the nonparametric Kruskal-Wallis rank-sum test was used to determine the interaction effects between the feedback use groups and the baseline values. Where significant differences among the groups could be determined, post hoc pairwise comparisons using the Wilcoxon signed-rank sum test were performed.

The resulting *P* values were adjusted by using the multiple testing correction method of Benjamini and Hochberg [31]. The η^2^ value based on the H-statistic was used as a measure of the effect size. A small effect is indicated by values <0.06, a moderate effect is indicated by values between 0.06 and 0.14, and a large effect is indicated by values ≥0.14 [30].

## Results

### Use Adherence and Feedback Use Groups

The descriptive statistics of the feedback use groups are shown in Table 1 as mean values and SD unless otherwise stated.

**Figure 3:**
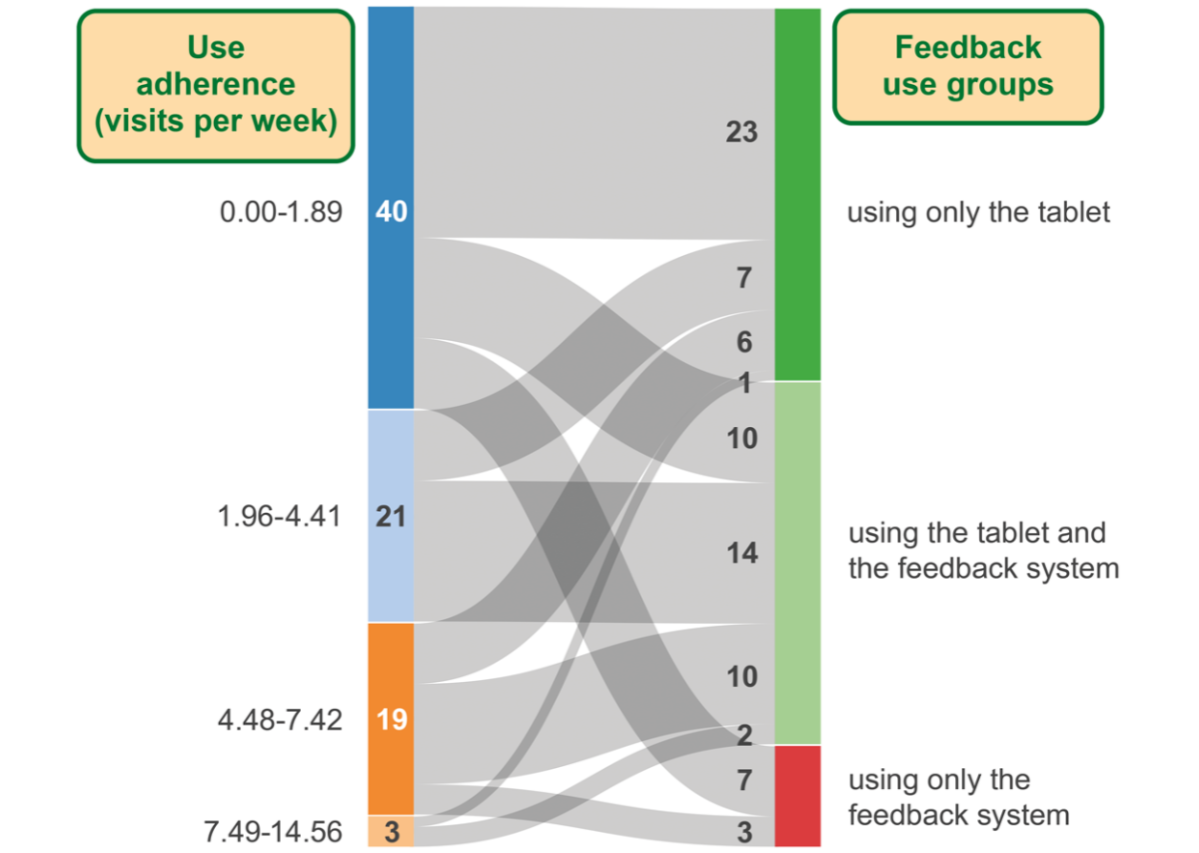
Sankey diagram showing the user flows of user types to the feedback use groups (only tablet-based exercise module used, tablet-based and feedback system–based exercise modules used, and only feedback system–based exercise module used) of the investigated 83 study participants.

Of the 83 users, 37 (45%) users exercised with the tablet-based exercise module without live feedback, and 36 (43%) users trained additionally with the feedback system. The remaining 12% (10/83) of participants used only the live feedback system to exercise. Of the 83 users, 4 user type clusters were determined: 40 (48%) users with up to 1.89 visits per week, 21 (25%) users with 1.96 to 4.41 visits per week, 19 (23%) users with 4.48 to 7.42 visits per week, and 3 (4%) users with 7.49 to 14.56 visits per week. Their distribution to the feedback use groups is shown in the Sankey diagram in Figure 3.

The use adherence of the first cluster with 48% (40/83) of the users did not meet the required 2 visits per week upper limit. Three-quarters of the users in this user type used only 1 of the 2 exercise modules, whereas 25% (10/40) used the tablet and the feedback system. The feedback use groups were adapted by rejecting this user type with 40 users, resulting in a total of 43 users. The descriptive statistics of the adapted feedback use groups are presented in Table 2. Looking at the distribution of the feedback use groups considering the use adherence, 40% (17/43) of the remaining users used either one of the exercise modules, and 60% (26/43) used the feedback system–based exercise module in addition to the tablet-based exercise module.

Investigating the workout information, on average 51% (868/1702) and 35% (596/1702) of the workouts in the feedback use group using the tablet and the feedback system were selected as 20-minute and 30-minute workouts, respectively. In comparison, users who exercised with only the tablet-based exercise module chose the 10-minute workout more often on average. In total, 1064 workouts were completed in the feedback use group using only the tablet, 1702 in the feedback use group using the tablet and the feedback system, and 242 workouts were completed in the feedback use group using only the feedback system.

**Table 1:** Descriptive characteristics of the baseline and feedback use groups without considering use adherence, including functional performance test results from baseline data and preintervention data for the feedback use groups (N=83).

Feedback use group	Baseline		Using only the tablet			Using the tablet and the feedback system			Using only the feedback system	
	Values, n (%)	Values, mean (SD)	Values, n (%)	Values, mean (SD)	Values, n (%)		Values, mean (SD)	Values, n (%)		Values, mean (SD)
Age (years)	83 (100)	66.2 (2.3)	37 (45)	65.9 (2.3)	36 (43)		66.3 (2.2)	10 (12)		66.8 (2.8)
Female	67 (81)	N/A^a^	30 (36)	N/A	30 (36)		N/A	7 (8)		N/A
Male	16 (19)	N/A	7 (8)	N/A	6 (7)		N/A	3 (4)		N/A
Workout duration (hours)	—^b^	—	37 (45)	12.7 (15.7)	36 (43)		24.9 (18.5)	10 (12)		11.2 (13.7)
Workout duration with feedback (hours)	—	—	NR^c^	NR	36 (43)		14.1 (16.7)	10 (12)		11.2 (13.7)
Number of completed workouts	—	—	37 (45)	28.8 (32.2)	36 (43)		47.3 (33.7)	10 (12)		24.2 (29.9)
10-minute workouts (%)	—	—	37 (45)	44 (36)	36 (43)		14 (14)	10 (12)		51 (46)
20-minute workouts (%)	—	—	37 (45)	32 (31)	36 (43)		51 (28)	10 (12)		42 (42)
30-minute workouts (%)	—	—	37 (45)	24 (32)	36 (43)		35 (29)	10 (12)		6 (12)
UPS^d^ maximum (seconds)	83 (100)	50.8 (16.2)	37 (45)	49.7 (17.7)	36 (43)		56.6 (9.0)	9 (11)		51.4 (15.9)
UPS left leg maximum (seconds)	82 (99)	45.1 (19.9)	37 (45)	47.4 (19.5)	36 (43)		51.7 (16.5)	9 (11)		47.3 (19.1)
UPS right leg maximum (seconds)	83 (100)	47.9 (18.3)	37 (45)	45.6 (18.9)	36 (43)		56.6 (9.0)	9 (11)		50.2 (15.9)
30-second CRT^e^ (chair rises)	80 (96)	16.0 (4.7)	37 (45)	15.3 (3.5)	36 (43)		17.6 (4.5)	9 (11)		15.6 (3.0)
YBT^f^ left leg composite score	71 (86)	81.7 (11.2)	36 (43)	79.6 (13.4)	36 (43)		85.9 (12.1)	8 (10)		81.2 (10.9)
YBT right leg composite score	71 (86)	80.4 (13.2)	36 (43)	78.7 (12.8)	36 (43)		85.2 (8.7)	8 (10)		82.2 (10.7)
YBT leg symmetry	71 (86)	3.9 (6.2)	36 (43)	3.5 (2.4)	36 (43)		3.4 (4.6)	8 (10)		2.2 (2.4)
HS^g^ left leg	79 (95)	2.2 (0.6)	37 (45)	2.1 (0.7)	36 (43)		2.3 (0.6)	8 (10)		2.1 (0.4)
HS right leg	79 (95)	2.1 (0.7)	37 (45)	2.1 (0.7)	36 (43)		2.3 (0.7)	8 (10)		2.3 (0.5)

^a^N/A: not applicable (no mean or SD values for sex categories applicable).

^b^Not available since baseline group did not receive any of the exercise modules.

^c^NR: not reported (not available since feedback use group using only the tablet did not perform exercises with the feedback system).

^d^UPS: unipedal stance.

^e^CRT: chair rise test.

^f^YBT: Y-balance test.

^g^HS: hurdle step.

**Table 2:** Descriptive characteristics of the feedback use groups considering use adherence (from 0.28 visits per day), including functional performance test results from preintervention data for the feedback use groups (N=43).

Feedback use group	Using only the tablet		Using the tablet and the feedback system			Using only the feedback system	
	Values, n (%)	Values, mean (SD)	Values, n (%)	Values, mean (SD)	Values, n (%)		Values, mean (SD)
Age (years)	14 (33)	64.9 (1.0)	26 (60)	66.0 (2.3)	3 (7)		66.0 (1.0)
Female	13 (30)	N/A^a^	22 (51)	N/A	3 (7)		N/A
Male	1 (2)	N/A	4 (9)	N/A	0 (0)		N/A
Workout duration (hours)	14 (33)	27.1 (17.1)	26 (60)	31.5 (17.7)	3 (7)		28.7 (6.7)
Workout duration with feedback (hours)	—^b^	—	26 (60)	17.5 (18.5)	3 (7)		28.7 (6.7)
Number of completed workouts	14 (33)	59.6 (32.9)	26 (60)	60.0 (31.2)	3 (7)		63.0 (20.4)
10-minute workouts (%)	14 (33)	34 (32)	26 (60)	15 (16)	3 (7)		4 (2)
20-minute workouts (%)	14 (33)	33 (28)	26 (60)	51 (28)	3 (7)		95 (2)
30-minute workouts (%)	14 (33)	33 (35)	26 (60)	35 (29)	3 (7)		1 (1)
UPS^c^ maximum (seconds)	14 (33)	59.0 (3.7)	26 (60)	58.0 (6.4)	3 (7)		60.0 (0.0)
UPS left leg maximum (seconds)	14 (33)	57.7 (5.9)	26 (60)	53.7 (15.2)	3 (7)		60.0 (0.0)
UPS right leg maximum (seconds)	14 (33)	51.9 (14.1)	26 (60)	57.8 (6.5)	3 (7)		60.0 (0.0)
30-second CRT^d^ (chair rises)	14 (33)	15.5 (3.3)	26 (60)	18.2 (4.6)	3 (7)		16.3 (3.3)
YBT^e^ left leg composite score	14 (33)	85.2 (6.1)	26 (60)	85.3 (13.4)	3 (7)		81.6 (8.2)
YBT right leg composite score	14 (33)	83.2 (7.6)	26 (60)	84.7 (9.0)	3 (7)		83.7 (5.8)
YBT leg symmetry	14 (33)	3.3 (2.6)	26 (60)	3.7 (5.1)	3 (7)		2.1 (2.4)
HS^f^ left leg	14 (33)	2.0 (0.7)	26 (60)	2.3 (0.6)	3 (7)		2.0 (0.0)
HS right leg	14 (33)	2.1 (0.5)	26 (60)	2.2 (30.7)	3 (7)		2.3 (0.6)

^a^N/A: not applicable (no mean or SD values for sex categories applicable).

^b^Not available since feedback use group using only the tablet did not perform exercises with the feedback system.

^c^UPS: unipedal stance.

^d^CRT: chair rise test.

^e^YBT: Y-balance test.

^f^HS: hurdle step.

### Effect of Exercise Modules on Balance and Leg Strength

#### Overview

The statistical results are presented in Table 3 without considering use adherence and in Table 4 considering use adherence.

**Table 3:** Statistical test results comparing the change of functional assessment results between baseline and feedback use groups without considering use adherence (N=83).

Feedback use group	Baseline			Using only the tablet			Using the tablet and the feedback system			Using only the feedback system			*P* value		Effect size
Functional performance change	Values, n (%)	Values, mean (SD)	Values, n (%)		Values, mean (SD)	Values, n (%)		Values, mean (SD)	Values, n (%)		Values, mean (SD)				
ΔUnipedal stance maximum	82 (99)	2.3 (8.5)^a^	27 (33)		4.4 (22.6)	31 (37)		1.9 (11.6)	4 (5)		8.8 (14.4)	.62		<0.06	
ΔUnipedal stance left leg maximum	82 (99)	4.2 (13.4)^a^	27 (33)		1.9 (27.4)	31 (37)		5.6 (19.4)	4 (5)		6.3 (39.3)	.94		<0.06	
ΔUnipedal stance right leg maximum	82 (99)	3.2 (10.4)^a^	27 (33)		7.2 (28.5)	31 (37)		2.0 (11.7)	4 (5)		11.3 (13.1)	.23		<0.06	
Δ30-second chair rise test	80 (96)	0.4 (2.7)^a^	26 (31)		5.8 (6.3)^a,b^	31 (37)		2.3 (5.9)^a^	5 (6)		6.3 (3.6)^b^	<.001^c^		0.18^d^	
ΔY-balance test left leg	71 (86)	0.7 (9.6)^a^	24 (29)		7.1 (13.7)^a^	31 (37)		−1.4 (14.1)^e^	4 (5)		15.2 (12.6)^b,f^	.01^c^		0.07^g^	
ΔY-balance test right leg	71 (86)	1.81 (10.1)^a^	24 (29)		6.2 (12.2)^a^	31 (37)		−2.0 (10.5)	4 (5)		10.2 (12.6)	.04^c^		<0.06	
ΔY-balance test symmetry	71 (86)	−0.7 (7.1)	24 (29)		−1.2 (3.3)	31 (37)		−0.3 (6.0)	4 (5)		−0.4 (2.8)	.42		<0.06	
ΔHurdle step left leg	79 (95)	0.0 (0.7)	25 (30)		−0.2 (0.9)	31 (37)		−0.3 (0.8)^a^	4 (5)		0.0 (1.4)	.29		<0.06	
ΔHurdle step right leg	79 (95)	0.1 (0.7)	25 (30)		−0.1 (0.8)	31 (37)		−0.3 (0.8)^a^	4 (5)		0.0 (1.4)	.11		<0.06	

^a^Significance within groups.

^b^Significant in comparison with the baseline group.

^c^Significance level α<.05.

^d^Large effect ≥0.14.

^e^Significant in comparison with the feedback use group using only the tablet.

^f^Significant in comparison with the feedback use group using the tablet and the feedback system.

^g^Moderate effect between 0.06 and 0.14.

**Table 4:** Statistical test results comparing the change of functional assessment results between baseline and feedback use groups considering use adherence (N=83).

Feedback use group	Baseline		Using only the tablet		Using the tablet and the feedback system		Using only the feedback system		*P* value	Effect size
Functional performance change	Values, n (%)	Values, mean (SD)	Values, n (%)	Values, mean (SD)	Values, n (%)	Values, mean (SD)	Values, n (%)	Values, mean (SD)		
ΔUnipedal stance maximum	82 (99)	3.0 (8.5)^a^	9 (11)	−10.9 (20.7)	22 (27)	0.7 (8.5)	1 (1)	0.0	.17	<0.06
ΔUnipedal stance left leg maximum	82 (99)	4.8 (13.5)^a^	9 (11)	−15.9 (22.8)	22 (27)	3.3 (18.2)	1 (1)	−47.0	.02^b^	0.07^c^
ΔUnipedal stance right leg maximum	82 (99)	3.9 (10.4)^a^	9 (11)	−4.1 (35.0)	22 (27)	0.8 (8.6)	1 (1)	0.0	.90	<0.06
Δ30-second chair rise test	80 (96)	0.4 (2.7)	9 (11)	3.8 (4.5)^a^	22 (27)	2.8 (6.2)^a^	1 (1)	6.5	.01^b^	0.07^c^
ΔY-balance test left leg	71 (86)	0.7 (9.6)^a^	7 (8)	2.6 (11.1)	22 (27)	−1.2 (15.8)	1 (1)	2.7	.33	<0.06
ΔY-balance test right leg	71 (86)	1.8 (10.1)	7 (8)	2.9 (10.2)	22 (27)	−2.7 (12.0)	1 (1)	−5.0	.20	<0.06
ΔY-balance test symmetry	71 (86)	−0.7 (7.1)	7 (8)	−0.2 (3.7)	22 (27)	−1.2 (6.0)	1 (1)	−1.9	.91	<0.06
ΔHurdle step left leg	79 (95)	0.0 (0.7)	8 (10)	−0.1 (0.8)	22 (27)	−0.4 (0.8)	1 (1)	0.0	.30	<0.06
ΔHurdle step right leg	79 (95)	0.1 (0.7)	8 (10)	−0.3 (0.7)	22 (27)	−0.3 (0.7)	1 (1)	0.0	.17	<0.06

^a^Significance within groups.

^b^Significance level α<.05.

^c^Moderate effect between 0.06 and 0.14.

#### Test Participation

The participants were able to withdraw themselves from performing 1 or all of the tests at any time. Several participants did not want to conduct single tests on site, although they would have been able to do so. The data availability of the functional performance assessments for each participant (ie, their test participation) varied depending on time point and type of test. The number of functional performance assessments conducted increased from basis to preintervention measurements: the number of participants who conducted the 30-second CRT increased from 96% (80/83) to 99% (82/83), YBT participation increased from 86% (71/83) to 96% (80/83), and HS test participation increased from 95% (79/83) to 98% (81/83). UPS test participation remained almost steady at approximately 99% (82/83) between baseline and preintervention data availability. In contrast, more preintervention than postintervention data on functional performance were available: the UPS test and CRT participation dropped from 99% (82/83) to 75% (62/83), the YBT participation dropped from 96% (80/83) to 71% (59/83), and the HS test data availability decreased from 98% (81/83) to 72% (60/83).

The reasons for the differences in participation were mainly scheduling problems because of the tight test schedule. If a person was late and, therefore, the test was only possible for a shorter time, there was no possibility to reschedule. Furthermore, the participants had the option of refusing to take a test. At the end of the intervention, not all study participants were convinced that they had benefited from the tests as the system simply had to be returned. Another less common reason was the weather. Temperatures were not always optimal for testing; thus, the participants were concerned about circulatory problems. Again, because of the tight schedule, no alternative date could be offered by the test team.

#### Not Considering Use Adherence

Significant improvements and interaction effects were mainly found for leg strength and dynamic balance when comparing the feedback use groups and the baseline difference.

The average static balance changes (UPS) were not different between the groups. Within the baseline group, the static balance (UPS) improved between basis and preintervention (*P*<.001).

The leg strength improvement of the 3 feedback use groups was significant in comparison with the baseline difference (*P*<.001), with a large effect size of 0.179. Pairwise comparisons determined that participants using either of the 2 exercise modules improved in leg strength compared with the baseline difference (*P*<.001 for feedback use group using only the tablet, and *P*=.01 for the feedback use group using only the feedback system). Furthermore, within the baseline difference (*P*=.03), the feedback use group using only the tablet (*P*<.001), and the feedback use group using the tablet and the feedback system (*P*=.02), the average leg strength comparing pre- and postintervention values was significant.

The dynamic balance improvement of the left leg (YBT left leg composite score) for the 2 feedback use groups (1) using only the tablet and (2) using only the feedback system was significant compared with the baseline difference, whereas it slightly decreased for feedback use group using the tablet and the feedback system in comparison with the baseline (*P*=.006; moderate effect size of 0.074). The pairwise comparison showed an improvement of dynamic balance change for the feedback use group using only the feedback system related to the baseline difference (*P*=.03) and to the feedback use group using the tablet and the feedback system (*P*=.03). The slight dynamic balance decrease in the left leg of the feedback use group using the tablet and the feedback system was significant compared with that of the feedback use group using only the tablet (*P*=.03).

Although the dynamic balance differences of the right leg were significant between the groups (*P*=.04; small effect size of 0.043), the pairwise comparisons were not significant. Dynamic balance comparing pre- and postintervention measures improved within the feedback use group using only the tablet (YBT of the right and the left legs, *P*=.02). Within the baseline difference, the dynamic balance performance improved comparing baseline and preintervention values (YBT of left and right leg, *P*=.01).

Comparing the pre- and postintervention values within the feedback use group using the tablet and the feedback system, the movement quality slightly decreased on average (HS left leg *P=*.04 and HS right leg *P*=.03). No further significance was observed.

#### Considering Use Adherence

When including only users who used the exercise modules at the WHO-recommended training frequency (Table 4), the baseline group showed significant improvement in static balance (UPS, *P*<.001) and dynamic balance of the left leg (YBT left leg, *P*=.005). Another within-group improvement in leg strength was identified in the feedback use group using only the tablet (*P*=.02) and in the feedback use group using the tablet and the feedback system (*P*=.04). The feedback use groups (using only the tablet, using the tablet and the feedback system, using only the feedback system) achieved, on average, more chair rises in 30 seconds than the baseline group (*P*=.01; moderate effect size of 0.073). In addition, the static balance of the left leg (UPS left leg maximum) of the feedback use group using the tablet and the feedback system and the baseline group improved in comparison with the feedback use groups using only the tablet and only the feedback system (*P*=.02; moderate effect size of 0.065). Pairwise comparisons did not reveal any further significance.

## Discussion

### Principal Findings

The purpose of this study was to assess the influence of digital exercise modules of a multimodular solution on balance and leg strength. As 2 digital exercise modules were administered within the study, use adherence to these 2 modules was considered to identify the users who met the recommended exercise frequency according to the WHO. To the best of our knowledge, this is the first study to evaluate the influence of single modules of a multimodular AAL solution on balance and leg strength considering use adherence. When considering the use adherence, a tendency toward a positive influence on leg strength was found for participants using the tablet-based or additionally using the feedback system–based exercise module but not between the feedback use groups. Without considering the use adherence to the digital home training, more positive effects were indicated; however, these are more likely to be induced by the other modules of the multimodular AAL solution or external influences than the digital exercise modules. Therefore, use adherence should be considered in the future for functional performance assessments of multimodular physical activity–promoting applications.

In this study, use adherence indicated that 52% (43/83) of the digital home training users were able to meet the WHO-recommended training frequency. This is almost twice the number of Austrian men and women aged 45 to 64 years who reported reaching the WHO-recommended frequency for muscle strengthening in 2019 (27.1% and 26.7%, respectively) [32].

Nevertheless, 1 cluster of user types was unable to comply with the training frequency recommended by the WHO. Previous research investigated the adherence to the modules of a multidomain lifestyle training, including cognitive training, nutrition, and exercise, provided on a tablet [33]. Although their participants mainly used the cognitive training module, they reported that the exercise module lacked diversity, challenges, and progression. The reasons in the fit4AAL study require further investigation; for example, if the study participants who did not meet the WHO-recommended training frequency used other modules more than the exercise modules and why.

However, the additional use of the feedback system tended to support the users to maintain the WHO-recommended training frequency and showed higher exercise doses. This is in alignment with the findings of the review by Brickwood et al [34], who showed that empowering technologies such as the commonly available activity trackers positively influenced physical activity participation.

The administered training program has already shown a positive influence on functional performance in older women in the first trial phase [19]. Nevertheless, the extent to which the choice of technology influences these effects has not yet been investigated. Exercise programs at home have already been proven to improve balance and reduce fall reduction rates in older adults [35-37].

Although the results of the analysis without considering use adherence confirmed the improvement in balance and leg strength, a different picture emerges when considering the use adherence: the improvement of leg strength remained within the feedback use groups using only the tablet and using the tablet and the feedback system. Improvements for the static balance only remained within the baseline group. Although the exercise modules improved the leg strength, a mean decrease in balance performance was observed. The decrease in static balance in the feedback use group using only the tablet was remarkable, whereas, in the feedback use group using the tablet and the feedback system, the static balance of the left leg remained or even improved.

This could be explained by the fact that the balance of the left leg could be increased by the intervention compared with the more likely dominant right leg. Moreover, digital exercise modules relying on tablet-based solutions without any training feedback might, on average, negatively influence the static balance of the participants. This strengthens the findings on slight reductions of static balance capabilities of regular app-using test groups in the course of multimonth studies [38]. Comparing the average pre- and postintervention assessment differences, all feedback use groups almost maintained their functional performance levels. Pairwise comparisons did not show any significance when considering the use adherence.

A possible explanation for the missing pairwise effects between the feedback use groups and the baseline could be that the functional performance test results of the baseline and preintervention assessments of the investigated participants were already within the age-appropriate norm values. Our sample of older adults was anything but fragile: the users were within and even beyond the norm values of their age group. For example, the minimum mean values of 15.3 (SD 3.5) chair rises (Table 1) already exceeded the norm values of 13.5 (SD 3.5) chair rises for the 30-second CRT for women aged 65 to 69 years [39]. In addition, the minimum range of 49.7 (SD 17.7) seconds of the UPS (Table 1) exceeded the age-appropriate norm value of 32.1 (SD 16.2) seconds [24]. Hence, the study participants were exceptionally fit. A focus on maintenance or even improvement of the functional performance values would require updated norm values of the age group and the region they are coming from. In Austria, the difference in complying with the WHO recommendations on physical activity varies between provinces [32]. Future research should investigate sample groups with different fitness levels in different age groups.

Moreover, a study design with groups using both exercise modules versus using only one of them with sufficient group size would be beneficial. Although a positive influence within the feedback use groups in leg strength was identified, this should be verified with a larger sample size separated into exercise module group, nondigital intervention group, and control group for more sufficient effect sizes [37,40].

In summary, the additional use of the feedback system–based exercise module might not yet verify the improvement of the functional performance in leg strength and balance of older adults who were trained at least twice a week. Nevertheless, the digital exercise modules of a multimodular AAL solution showed a tendency to positively maintain and influence the already exceptionally high functional performance in older adults. Moreover, the use of additional use of empowering technologies could support users to achieve their training goals, keeping the known challenges such as usability and technology acceptance in mind.

As 2 digital exercise modules within a multimodular AAL solution were used within an 18-week intervention, this study is the first attempt to investigate functional performance outcomes considering the use adherence of single modules, except the entire AAL solution, in older adults. The main recommendations for future field trials, in particular, AAL field studies, is to focus on modules and consider or rather nudge the voluntary use. Our intervention group was able to use all modules instead of only particular modules. This intended and resulted in maintaining high adherence and engagement with the system. As practical implications for further field studies aiming to investigate the influence of particular modules on selected markers of functional performance of the participants, it would be beneficial to use already evaluated modules in the AAL study or to test newly developed exercise modules in a prefield trial with friendly users independent from other modules. Hence, two main challenges for the analysis procedure were identified: (1) the multimodularity of solutions, which requires user type clustering to identify whether and how often the particular modules were used, and (2) the selection of specific intervention measures describing the influence of particular modules.

### Limitations

A limitation of this study is that no paper-based interventions were evaluated. Owing to the separation into feedback use groups, the sample size for the statistical analysis decreased; thus, the statistical power and generalizability were reduced. In future studies, more users should be included to reduce the risk of small sample sizes when clustering the users. Furthermore, additional motivation strategies to keep users engaged with the system, as well as the functional performance tests, have to be considered. Although the recruitment targeted a balanced study sample, more women than men applied for the study. For generalizability, not only a larger sample size but also a sex-balanced sample size would be beneficial for analysis by gender with sufficient statistical power. Whether the system or study attracted fewer men than women was not investigated.

### Conclusions

Although there are variations in the use of the digital exercise modules, the additional use of a feedback system in the multimodular AAL solution of the fit4AAL project positively influences use adherence and improvements in leg strength within feedback use groups. Thus, involving various empowering technologies to keep people engaged in their unsupervised digital home training can be recommended for active aging. The feedback system in this study integrates a split-screen view and posture tracking for repetition counting and exercise monitoring for selected exercises. In the future, more movement quality–monitoring functionalities, integrating further trackers or even on-body sensor networks of, for example, smart textiles and smartwatches, could be considered. To further justify the positive influence of the additional feedback system–based exercise module on functional performance, a different study design must be considered with, for example, different age groups, because of different technology savvy and functional performance levels. The participants of this study were exceptionally fit compared with the norm values of their age groups. Whether the digital exercise modules in multimodular AAL solutions can generate more benefit in establishing a home training routine in older adults has to be clarified in future longitudinal studies.

## Abbreviations

AAL: Active and Assisted Living
CRT: chair rise test
HS: hurdle step
ICT: Information and Communication Technology
UPS: unipedal stance
WHO: World Health Organization
YBT: Y-balance test
